# Nutritional Deficiencies and Subfertility: A Comprehensive Review of Current Evidence

**DOI:** 10.7759/cureus.66477

**Published:** 2024-08-08

**Authors:** Swasti Shukla, Deepti Shrivastava

**Affiliations:** 1 Obstetrics and Gynecology, Jawaharlal Nehru Medical College, Datta Meghe Institute of Higher Education and Research, Wardha, IND

**Keywords:** clinical evidence, micronutrients, fertility interventions, reproductive health, nutritional deficiencies, subfertility

## Abstract

Subfertility, a condition marked by a reduced capacity to conceive naturally, affects a significant proportion of couples globally. Nutrition is a fundamental aspect of reproductive health, with various nutrients essential in maintaining optimal reproductive function. This comprehensive review explores the intricate relationship between nutritional deficiencies and subfertility. It examines key micronutrients such as vitamins D, E, C, and B12, as well as minerals such as zinc, iron, selenium, and magnesium, and their impacts on fertility. The review also considers macronutrients and the importance of a balanced diet in supporting reproductive health. Drawing on an extensive body of clinical evidence and studies, this review highlights how deficiencies in these nutrients can lead to hormonal imbalances, impaired gametogenesis, and suboptimal pregnancy outcomes. It discusses the efficacy of nutritional interventions, including dietary supplements and lifestyle modifications, in improving fertility. Furthermore, it addresses the emerging research on personalized nutrition and its potential to enhance reproductive outcomes. The review underscores the necessity for healthcare providers to assess and address the nutritional status of patients with subfertility. It provides practical recommendations for developing nutritional plans, counseling patients, and integrating nutritional interventions into fertility treatments. By offering a comprehensive synthesis of current evidence, this review aims to inform clinical practice and promote further research into the role of nutrition in enhancing fertility.

## Introduction and background

Subfertility is a condition characterized by a reduced ability to conceive naturally. Unlike infertility, which is the inability to conceive after one year of regular unprotected intercourse, subfertility indicates a lower-than-average likelihood of achieving pregnancy within the same timeframe [1]. Subfertility affects approximately 10-15% of couples worldwide, making it a significant public health concern. The condition can result from a variety of factors, including, but not limited to, hormonal imbalances, structural abnormalities, lifestyle factors, and nutritional deficiencies [2].

Nutrition plays a crucial role in maintaining overall health, including reproductive health. Adequate intake of essential nutrients is vital for the proper functioning of the reproductive system in both men and women [3]. Nutritional deficiencies can lead to hormonal imbalances, poor sperm and egg quality, and impaired fetal development. Vitamins, minerals, and other nutrients support processes such as hormone production, ovulation, and sperm motility. Given the complex interplay between nutrition and fertility, addressing nutritional deficiencies is a key component of improving reproductive outcomes [4].

This comprehensive review aims to synthesize current evidence on the relationship between nutritional deficiencies and subfertility. It seeks to explore the impact of various micronutrient and macronutrient deficiencies on reproductive health, examine clinical studies and interventions, and provide practical recommendations for clinicians. By highlighting the critical role of nutrition in fertility, this review aims to inform and guide healthcare professionals in the development of effective nutritional strategies to support couples experiencing subfertility.

## Review

Overview of subfertility

Definition and Distinction From Infertility

Subfertility is defined as a reduced ability to conceive, marked by an extended period of unsuccessful attempts to become pregnant. Infertility, on the other hand, refers to the inability to conceive after one year of trying (or six months for women over 35) [5]. The primary distinction between the two conditions lies in the duration of unsuccessful attempts and the potential for future pregnancies. Couples who are subfertile may still have a chance of achieving pregnancy naturally, although it may take longer. In contrast, infertility suggests a more severe reproductive issue, where the likelihood of achieving pregnancy without medical intervention is significantly reduced. Understanding these definitions is essential for couples trying to conceive, as it helps set realistic expectations and informs the selection of appropriate medical evaluations and treatments [6].

Causes and Risk Factors

**Figure 1:**
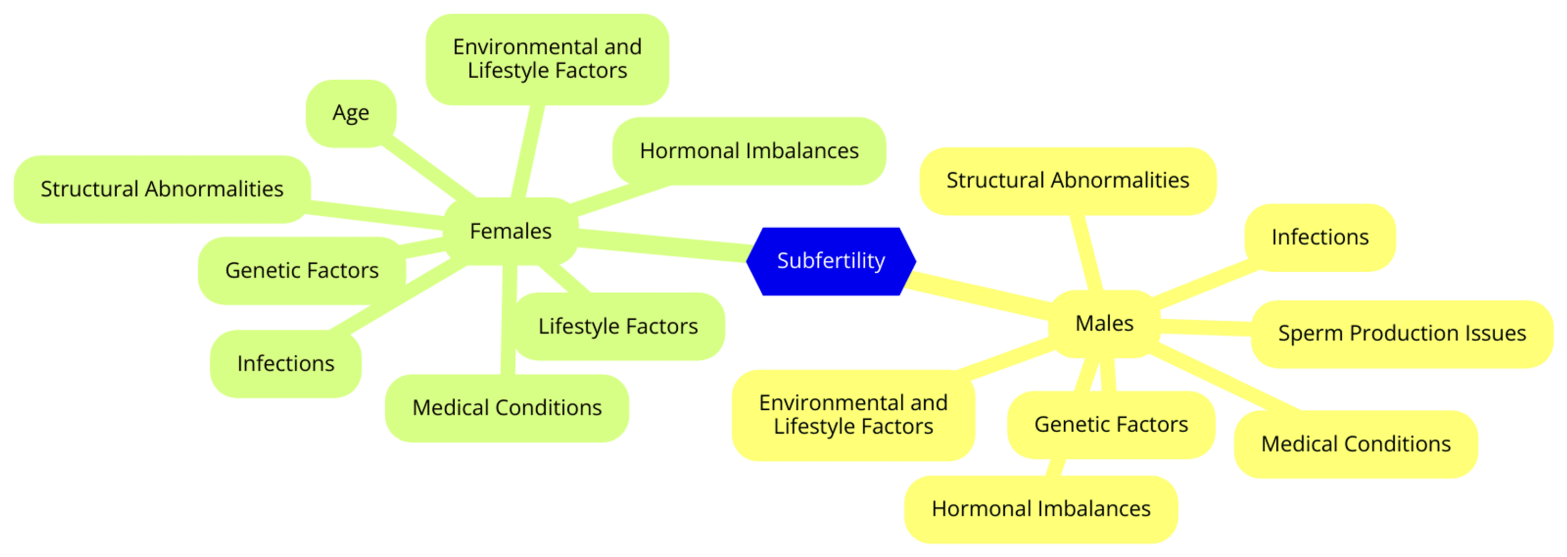
Causes and risk factors of subfertility in males and females Image Credit: Dr Swasti Shukla

Male factor subfertility is a significant contributor to infertility, estimated to play a role in approximately 50% of couples experiencing subfertility. Major causes include issues with semen quality, such as low sperm count (oligospermia), reduced sperm motility (asthenospermia), and abnormal sperm morphology, all of which can severely impact fertility. Hormonal imbalances, including disruptions in testosterone and other hormone levels, can affect sperm production. Additionally, genetic factors, such as chromosomal abnormalities, can lead to infertility by impacting sperm development and function [7]. Environmental and lifestyle factors also play a crucial role. Exposure to toxins such as pesticides and heavy metals, high temperatures, smoking, excessive alcohol consumption, obesity, and poor nutrition have all been linked to decreased sperm quality and fertility. Furthermore, medical conditions such as varicocele (enlargement of veins within the scrotum), infections, and chronic diseases can contribute to male subfertility [8]. Female subfertility can arise from a variety of issues. Ovulation disorders, such as polycystic ovary syndrome (PCOS) and hormonal imbalances, can prevent regular ovulation, making conception difficult. Structural abnormalities, including blocked fallopian tubes (often caused by infections) or endometriosis (uterine abnormalities or fibroids), can hinder fertilization or embryo implantation [9]. Age is another significant factor, as female fertility declines with age, especially after 35, due to a reduction in both the quantity and quality of eggs. Endometriosis can further complicate fertility by causing inflammation and scarring that disrupt reproductive anatomy. Lifestyle factors, similar to those affecting men, such as obesity, smoking, and excessive alcohol intake, can also negatively impact female fertility [10]. In many cases, both partners may contribute to subfertility, complicating diagnosis and treatment. Age is a critical factor, as declining fertility in both partners with age increases the likelihood of subfertility, with potential sperm quality issues in men and reduced fertility in women. Genetic compatibility is another consideration, as genetic factors affecting one partner can influence the reproductive success of the other, presenting challenges in achieving pregnancy [11]. Shared lifestyle factors can also be influential, as couples may adopt similar habits that adversely affect fertility, such as poor diet, lack of exercise, and substance use. Additionally, psychological stress related to subfertility can affect both partners, potentially exacerbating difficulties in their efforts to conceive [12]. Causes and risk factors of subfertility in males and females are shown in Figure 1.

Impact of Subfertility on Individuals and Couples

Subfertility can profoundly impact individuals and couples, influencing their emotional, psychological, and social well-being. Those dealing with subfertility often experience significant distress, anxiety, and depression. The emotional toll may manifest as sadness, anger, frustration, and diminished self-esteem. The stress of not being able to conceive can create feelings of helplessness and a perceived loss of control over one's life and future. Couples may cope with this emotional burden differently, which can lead to misunderstandings and resentment [13]. One partner might feel overwhelmed by the treatment process, while the other may struggle to articulate their feelings, resulting in emotional distance and isolation. Additionally, many couples undergoing fertility treatments may withdraw from social interactions, particularly with friends and family who have children, exacerbating feelings of loneliness and despair [13-14]. For couples who already have children, the desire to provide siblings can create guilt and anxiety about balancing attention between existing children and the pursuit of additional family members. Seeking support from others who understand their struggles, whether through support groups or counseling, can be beneficial, as open communication about their experiences can help strengthen relationships and alleviate feelings of isolation [14]. Moreover, societal and cultural pressures can complicate the emotional landscape of subfertility. The stigma associated with infertility, especially in cultures where childbearing is highly valued, can lead to feelings of inadequacy and shame, particularly for women who may face additional scrutiny regarding their reproductive capabilities. The financial burden of fertility treatments can also contribute to stress, as many couples face substantial out-of-pocket expenses, leading to financial strain and anxiety about their future [15].

Key nutrients and their role in reproductive health

Micronutrients

Micronutrients, especially vitamins, are crucial for reproductive health. Vitamin D is key in regulating reproductive hormones, including estrogen and progesterone, and is essential for ovulation and menstrual regularity in women. For men, adequate vitamin D levels are associated with improved sperm motility and overall quality [16]. Additionally, sufficient vitamin D during pregnancy is linked to a reduced risk of complications such as gestational diabetes and preeclampsia. Sunlight is the primary source of vitamin D. Still, it can also be obtained from dietary sources such as fatty fish (e.g., salmon and mackerel), fortified dairy products, egg yolks, and mushrooms [16]. Vitamin E is a powerful antioxidant that protects reproductive cells from oxidative stress, which can negatively affect fertility. It is important for maintaining sperm membrane integrity and function, contributing to overall sperm health. Some studies suggest that vitamin E may alleviate premenstrual syndrome (PMS) symptoms and support regular menstrual cycles. Rich sources of vitamin E include nuts and seeds (particularly almonds and sunflower seeds), vegetable oils (such as sunflower and olive oil), spinach, and avocados [17]. Vitamin C, another antioxidant, helps protect sperm and egg cells from oxidative damage and supports the synthesis of hormones, including estrogen, which is vital for female fertility. Research has shown that vitamin C supplementation can enhance sperm motility and reduce DNA damage in sperm cells. The best sources of vitamin C are fruits and vegetables, particularly citrus fruits (such as oranges and lemons), strawberries, kiwi, bell peppers, and broccoli [18]. Folate (vitamin B9) is essential for DNA synthesis and repair, making it particularly important in early pregnancy to prevent neural tube defects. It also supports ovulation and may enhance fertility in women [19]. Vitamin B12 is crucial for red blood cell production and DNA synthesis; deficiency can lead to anemia and affect fertility. It is involved in hormone regulation and maintaining a healthy menstrual cycle. Folate is found in leafy greens, legumes, nuts, and fortified grains, while vitamin B12 is primarily present in animal products such as meat, fish, eggs, and dairy. Vegans may need to consider fortified foods or supplements to meet their vitamin B12 needs [19].

Minerals

Zinc is essential for male reproductive health, playing a critical role in the development and function of male reproductive organs, as well as in sperm production and maturation. Adequate zinc levels are necessary for maintaining testosterone levels, which are crucial for sexual health and fertility [20]. Zinc deficiency can result in decreased sperm quality and count, potentially leading to fertility issues. Key dietary sources of zinc include oysters, red meat, poultry, beans, and nuts. Including these foods can help ensure sufficient zinc intake and support overall reproductive function [20]. Iron is another vital mineral for reproductive health, particularly for women. It is essential for producing hemoglobin, the protein in red blood cells that carries oxygen throughout the body. Adequate iron levels are especially important for women due to the risk of anemia from menstrual blood loss [21]. Iron deficiency can lead to fatigue, weakness, and impaired fertility, making it crucial for women of childbearing age to monitor their iron intake. Iron-rich foods include lean meats, beans, spinach, and iron-fortified cereals. Incorporating these sources into the diet can help maintain optimal iron levels and support reproductive health [21]. Selenium, an important antioxidant, helps protect the body's cells, including reproductive cells, from oxidative damage. It is also critical for properly functioning the thyroid gland, which regulates hormones essential for fertility. Research suggests that selenium deficiency may be linked to reduced sperm motility in men and an increased risk of miscarriage in women. Individuals can include selenium-rich foods in their diet to support reproductive health, such as seafood, Brazil nuts, and organic meats [22]. Magnesium is involved in more than 300 enzymatic reactions and is crucial for hormone regulation. It plays a role in the development and function of the ovaries and testes, making it vital for reproductive health. Magnesium deficiency has been associated with complications during pregnancy, such as an increased risk of preeclampsia and gestational diabetes. Individuals should include magnesium-rich foods, such as green leafy vegetables, whole grains, nuts, and seeds, to promote reproductive health. Ensuring adequate magnesium intake supports reproductive systems and overall health [23].

Macronutrients

Macronutrients - proteins, fats, and carbohydrates - are fundamental components of our diet, each playing a critical role in overall health, including reproductive health. Additionally, while not classified as macronutrients, antioxidants protect cells from oxidative stress [24]. Proteins are vital for the growth and repair of tissues, including reproductive organs. Many reproductive hormones, such as insulin and gonadotropins, are proteins or peptides. Adequate protein intake in men is associated with improved sperm quality and motility. Key protein sources include lean meats, fish, eggs, dairy products, and plant-based options such as beans, lentils, tofu, nuts, and seeds [25]. Fats are essential for the production of sex hormones that regulate the menstrual cycle and fertility. They also facilitate the absorption of fat-soluble vitamins (A, D, E, and K), which are important for reproductive health. Healthy fats contribute to the structure of cell membranes, including those of reproductive cells. Sources of healthy fats include avocados, olive oil, nuts, seeds, and fatty fish. Limiting trans fats and saturated fats can help maintain overall health [26]. Carbohydrates are the body's primary energy source, supporting all bodily functions, including reproduction. Complex carbohydrates help maintain stable blood sugar levels, crucial for hormonal balance and reproductive health. High-fiber carbohydrates promote gut health and help regulate hormones. Whole grains, fruits, vegetables, legumes, nuts, and seeds are excellent sources of carbohydrates [27]. Antioxidants neutralize free radicals and reduce oxidative stress that can damage reproductive cells and tissues. Antioxidants such as vitamins C and E can enhance sperm quality and motility in men and support menstrual regularity and ovulation in women. Rich sources of antioxidants include fruits, vegetables, nuts, seeds, green tea, and dark chocolate [28]. A balanced intake of macronutrients and a rich supply of antioxidants is crucial for optimal reproductive health. A well-rounded diet incorporating these elements can enhance fertility and support overall well-being for both men and women. For individuals trying to conceive, focusing on nutrient-dense foods and maintaining a healthy lifestyle can significantly improve reproductive outcomes [4].

Nutritional deficiencies and their impact on subfertility

Vitamin Deficiencies

Vitamin deficiencies can significantly impact reproductive health and contribute to subfertility. Here is a detailed overview of specific vitamin deficiencies - vitamin D, vitamin E, vitamin C, and B vitamins (including B12 and folate) - and their effects on fertility [29]. Vitamin D is essential for various bodily functions, including immune response and bone health, and it plays a significant role in reproductive health. Deficiency in vitamin D has been linked to irregular menstrual cycles and conditions such as PCOS, which can impair ovulation [29]. Adequate vitamin D levels may enhance ovarian function and improve the chances of successful implantation. In men, low vitamin D levels are associated with poor sperm quality, including reduced motility and concentration, and may also affect testosterone levels, which are crucial for male fertility. Vitamin D can be obtained from sunlight exposure, fatty fish (such as salmon and mackerel), fortified foods (such as milk and orange juice), and supplements [29]. Vitamin E is a fat-soluble antioxidant that protects cells from oxidative stress and supports immune function. It is vital for maintaining healthy ovarian function and may aid in developing the corpus luteum, which is essential for hormone production. Deficiency in vitamin E can lead to reproductive issues and complications during pregnancy [30]. In men, adequate vitamin E levels are important for sperm health, as they help protect sperm from oxidative damage, which affects motility and overall fertility. Sources of vitamin E include nuts and seeds (such as almonds and sunflower seeds), vegetable oils (such as sunflower and olive oil), green leafy vegetables, and fortified cereals [30]. Vitamin C is a water-soluble vitamin known for its antioxidant properties and role in collagen synthesis. It is crucial for hormone production and may improve ovarian function. Vitamin C also supports the health of the endometrium, which is vital for embryo implantation [31]. In men, vitamin C is essential for sperm health as it protects sperm from oxidative stress and may improve motility. Supplementation has been shown to enhance sperm quality in individuals with low vitamin C levels. Good sources of vitamin C include citrus fruits (such as oranges and lemons), berries (such as strawberries and blueberries), kiwi, bell peppers, and broccoli [31]. B vitamins, particularly B12 and folate (B9), are crucial for DNA synthesis, red blood cell formation, and overall cellular function. Folate is essential for proper cell division and has been linked to improved ovulation and reduced risk of neural tube defects during pregnancy. Vitamin B12 deficiency can lead to menstrual irregularities and may impair fertility [32]. Vitamin B12 and folate are important for sperm production and health, with deficiencies potentially leading to poor sperm morphology and motility, affecting overall fertility. Vitamin B12 is found in animal products (such as meat, fish, and dairy) and fortified cereals, while folate is abundant in leafy greens (such as spinach and kale), legumes (such as beans and lentils), and fortified grains [32].

Mineral Deficiencies

Mineral deficiencies can profoundly impact reproductive health and contribute to subfertility in both men and women. Key minerals such as zinc, iron, selenium, and magnesium play crucial roles in reproductive functions, and deficiencies can lead to various fertility issues [33]. Zinc deficiency is particularly detrimental to reproductive health. In women, insufficient zinc levels can adversely affect the early stages of egg development, leading to reduced egg quality and impaired fertilization [34]. Zinc is essential for oocyte maturation, and its deficiency can result in smaller egg cells and issues with meiotic division, which is crucial for successful fertilization. In men, zinc deficiency is associated with poor sperm quality, including abnormalities in sperm morphology and motility. Additionally, low zinc levels can reduce testosterone production, which is critical for male reproductive health. Supplementing with zinc has been shown to enhance sperm quality and overall fertility outcomes [34]. Iron deficiency is another significant nutritional concern that can affect fertility. Low iron levels can lead to anemia in women, which is linked to irregular menstrual cycles and decreased ovulation [35]. Women with iron deficiency may experience longer times to conceive, as adequate iron levels are necessary for proper ovarian function and overall reproductive health. In men, iron deficiency can also impair fertility by affecting testosterone levels and sperm production. Anemia can reduce energy levels and overall health, which may indirectly impact reproductive capabilities [35]. Selenium is an essential trace element that plays a role in antioxidant defense and reproductive health. In women, selenium deficiency has been associated with impaired ovarian function and increased oxidative stress, which can negatively affect egg quality and fertility. For men, selenium is vital for sperm motility and overall sperm health. Studies indicate that low selenium levels can lead to decreased sperm quality and increased risks of infertility. Adequate selenium intake supports sperm production and function [36]. Magnesium is another mineral crucial for numerous biochemical processes, including reproductive health. In women, magnesium deficiency can disrupt hormonal balance, leading to irregular menstrual cycles and ovulatory issues. Magnesium synthesizes sex hormones, which are critical for ovulation and fertility. In men, magnesium is essential for testosterone production and sperm health. Low magnesium levels can result in reduced testosterone levels, negatively impacting sperm production and quality [33].

Effects of Combined Deficiencies

Combined nutritional deficiencies can have a profound impact on overall health and significantly affect subfertility in both men and women. The interplay between various nutrient deficiencies often exacerbates health issues, leading to more severe outcomes than single deficiencies alone. For example, deficiencies in vitamins A, C, and D and minerals such as zinc can impair immune function, making individuals more susceptible to infections. Combined deficiencies of iron, folate, and vitamin B12 can result in anemia, characterized by fatigue and decreased oxygen transport, which complicates reproductive health by affecting energy levels [37]. In women, such deficiencies can disrupt hormonal balance and ovulatory function. Folate is essential for DNA synthesis and repair, while iron deficiency can lead to anemia, reducing fertility due to insufficient oxygen delivery to reproductive organs. Additionally, vitamin D deficiency has been linked to conditions such as PCOS, which can affect ovulation [33]. In men, combined deficiencies can adversely affect sperm quality. Zinc and selenium are crucial for testosterone production and sperm motility, and deficiencies in these nutrients can lead to reduced sperm quality and quantity. Furthermore, low levels of vitamin D have been associated with lower testosterone levels, further hindering male fertility [38]. Omega-3 fatty acids, while not vitamins or minerals, are essential for sperm membrane integrity and function; their deficiency can further complicate fertility issues. Addressing these combined nutritional deficiencies through dietary changes and supplementation is vital for improving overall health and fertility outcomes. A balanced diet rich in essential nutrients is crucial for both men and women, aiming to optimize reproductive health. Preventive measures, including nutrition education and access to a diverse range of foods, can help mitigate the risks associated with these deficiencies [38].

Nutritional Interventions and Fertility

Dietary supplements can play a significant role in addressing nutritional deficiencies that may affect fertility. For men, nutrients such as zinc, selenium, folate, vitamin C, vitamin E, and omega-3 fatty acids are associated with improved sperm quality. Research indicates that these supplements can enhance sperm count, motility, and morphology in cases of male subfertility. However, specific supplements' efficacy and optimal dosages still require further investigation [39]. For women, folic acid supplementation is essential for those planning to conceive, as it helps prevent neural tube defects in the developing fetus. Vitamin D deficiency has also been linked to reduced fertility, suggesting supplementation may benefit some women. Other supplements, including antioxidants and probiotics, have also been studied for their potential positive effects on female fertility, but more robust evidence is needed to draw definitive conclusions. It is crucial to approach dietary supplements cautiously, as they are not well regulated, and the quality can vary significantly between products. Some supplements may interact with medications or lead to side effects, especially in high doses or in combination. Therefore, supplements should complement a healthy, balanced diet rather than replace it as the primary strategy for optimizing fertility [40]. In addition to supplements, diet and lifestyle changes are essential for enhancing fertility. A balanced diet rich in fruits, vegetables, whole grains, lean proteins, and healthy fats provides the essential nutrients for reproductive health. A Mediterranean-style diet, which includes ample dietary fiber, omega-3 fatty acids, and plant-based proteins, has positively impacted fertility outcomes for both men and women [41]. Regular physical activity is another vital component; it helps maintain a healthy weight, reduces stress, and improves overall well-being, contributing to better fertility. Weight management is particularly important, as both underweight and overweight conditions can disrupt hormonal balance and affect reproductive functions. Achieving and maintaining a healthy weight through a nutritious diet and regular exercise can significantly enhance fertility prospects [41]. Enhancing with additional nutrients or bioactive compounds, functional foods can also support fertility. These foods may include fortified cereals, probiotic-rich yogurts, and omega-3-enriched products [42]. Incorporating such foods into the diet can help address specific nutritional deficiencies and promote overall health. For example, foods rich in antioxidants can combat oxidative stress, which is linked to reduced fertility in both men and women. Including various functional foods can provide additional health benefits and support reproductive health [42]. Lastly, the consumption of alcohol and caffeine can significantly impact fertility. High alcohol intake has been associated with decreased fertility in both men and women. For men, excessive alcohol consumption can lead to lower testosterone levels and reduced sperm production. For women, alcohol can disrupt hormonal balance and affect ovulation [43]. Caffeine, while generally safe in moderate amounts, may also have negative effects on fertility when consumed in excess. Studies suggest that high caffeine intake may be linked to an increased risk of miscarriage and may affect the ability to conceive. Therefore, it is advisable for individuals trying to conceive to limit their intake of alcohol and caffeine to optimize fertility [43].

Clinical evidence and studies

The impact of nutritional deficiencies on male fertility has been extensively studied. Research indicates that diets low in essential nutrients, such as omega-3 fatty acids and antioxidants (vitamins E and C, selenium, zinc), and high in saturated and trans fats correlate with poor semen quality parameters, including reduced sperm count and motility [44]. Nutritional deficiencies can also lead to hormonal disruptions, particularly in obese individuals, where elevated estrogen levels are observed alongside decreased testosterone, luteinizing hormone (LH), and follicle-stimulating hormone (FSH) levels. This hormonal imbalance can further compromise spermatogenesis and overall fertility. Additionally, antioxidant deficiencies can increase oxidative stress, negatively impacting sperm DNA integrity and overall reproductive health. Studies show that men with higher intakes of vitamins C and E experience improved sperm quality and lower DNA fragmentation [44]. Nutritional interventions have shown promising results in enhancing male fertility outcomes. Adopting a diet rich in fruits, vegetables, whole grains, and lean proteins while reducing processed foods and trans fats has been associated with better sperm quality. For instance, a Mediterranean diet has been positively linked to improved semen parameters. Specific supplements, such as omega-3 fatty acids, coenzyme Q10, and antioxidants, have demonstrated benefits in enhancing sperm motility and reducing oxidative stress. Studies indicate that men taking vitamin C and E supplements have improved sperm motility and overall reproductive health [44]. Nutritional deficiencies also severely impact female reproductive health. Malnutrition and inadequate intake of proteins, vitamins, and minerals are linked to disrupted ovulatory cycles and reduced fertility. Conditions such as anorexia and bulimia are directly associated with infertility due to their impact on hormonal balance and ovulation. Poor nutrition can adversely affect oocyte maturation and embryonic development, increasing the risks of infertility and miscarriage. Studies suggest deficiencies in critical nutrients, including folate and vitamin D, may impair fertility by affecting gamete quality and implantation [37]. Nutritional interventions can enhance female fertility. A diet rich in omega-3 fatty acids, dietary fiber, and antioxidants has positively influenced female fertility. For instance, adherence to a Mediterranean diet has been associated with improved reproductive outcomes. Women of childbearing age are often advised to supplement with folic acid and monitor vitamin D levels, as these have been shown to improve fertility rates. Adequate iodine intake is also crucial, especially in populations with dietary restrictions [45]. Clinical trials and case studies have provided valuable insights into the relationship between nutrition and fertility. Various trials have explored the effects of dietary modifications and supplementation on fertility outcomes [46]. Studies examining the impact of antioxidant supplementation on sperm quality have yielded positive results, indicating improvements in motility and DNA integrity in men. Observational studies have documented cases in which dietary interventions significantly improved fertility parameters. For instance, couples who adopted healthier eating patterns reported higher success rates in conception and improved reproductive health outcomes [47].

Emerging research and future directions

Emerging research in nutrition and fertility is uncovering innovative approaches with the potential to significantly impact reproductive health. One key area of focus is the development of novel nutritional strategies. Recent studies highlight dietary patterns' critical role in fertility outcomes [48]. Diets high in trans fats, refined carbohydrates, and sugars are associated with adverse effects on fertility, while diets rich in whole foods such as the Mediterranean diet, which includes ample dietary fiber, omega-3 fatty acids, and plant-based proteins, show promising benefits for reproductive health. Researchers are also exploring the role of the gut microbiome in fertility, investigating how dietary choices may influence microbiota composition and, subsequently, infertility rates. Phytoestrogens, plant-derived compounds that may positively affect female fertility, are another area of interest, though further research is needed to clarify their impact [4]. Personalized nutrition is an emerging theme in fertility research, focusing on the potential for tailored dietary recommendations based on individual needs, genetic predispositions, and specific health conditions. This approach aims to enhance fertility outcomes by addressing unique dietary habits and nutrient requirements [49]. For example, combining dietary assessments with assisted reproductive technologies (ART) could optimize treatment efficacy. Studies suggest that understanding an individual’s specific dietary habits can lead to more effective interventions, including personalized supplementation strategies for essential nutrients such as folic acid, vitamin D, and iodine, which are vital for reproductive health [50]. Moreover, integrating nutritional interventions into fertility treatments is increasingly recognized as a vital component of comprehensive reproductive care. Healthcare providers are encouraged to offer evidence-based dietary guidance alongside traditional fertility treatments. Research indicates that dietary modifications can significantly influence the outcomes of ART procedures, such as in vitro fertilization (IVF) [51]. Nutritional interventions may improve embryo quality and enhance the likelihood of successful pregnancies. As a result, the future of fertility treatments may increasingly incorporate comprehensive nutritional strategies that address both dietary habits and individual health profiles, ultimately aiming to improve reproductive outcomes for couples facing infertility challenges [51].

Practical recommendations for clinicians

Assessing a patient's nutritional status is a critical initial step in addressing potential subfertility linked to nutritional deficiencies. Clinicians should take a detailed medical and dietary history to identify any risk factors or existing deficiencies [52]. A thorough physical examination can reveal clinical signs of nutrient deficiencies, such as pallor, Bitot's spots, pitting edema, goiter, and signs of wasting. Additionally, ordering laboratory tests to evaluate micronutrient levels, such as iron, folate, and vitamin D, provides valuable insights based on the patient’s history and examination findings. Anthropometric measurements, including body mass index (BMI) and mid-upper arm circumference (MUAC), can further assess overall nutritional status [53]. Once the nutritional status has been evaluated, clinicians should develop personalized dietary recommendations tailored to the patient’s needs. This involves considering individual food preferences, cultural backgrounds, and dietary restrictions [54]. The emphasis should be on promoting nutrient-dense whole foods, such as fruits, vegetables, whole grains, lean proteins, and healthy fats. When deficiencies are identified, appropriate supplements such as folic acid may be recommended to address these gaps for women planning to conceive. Written educational materials can reinforce counseling and help patients understand the importance of maintaining healthy eating patterns [54]. Effective counseling is essential for encouraging dietary changes that support fertility. Clinicians should explain the significance of good nutrition for fertility and pregnancy outcomes empathetically and non-judgmentally [55]. Offering specific examples of nutrient-rich foods and sharing healthy recipes can make dietary changes more achievable. Discussing cultural or religious dietary restrictions and working within those parameters is essential to developing a feasible plan. Clinicians should stress that while supplements can be beneficial in certain situations, they are not a substitute for a balanced and healthy diet [55]. Ongoing monitoring and adjustment of interventions are crucial to ensuring that dietary changes are effective and sustainable. Clinicians should schedule follow-up appointments to assess the patient’s progress and address any challenges with dietary modifications [56]. Periodic re-evaluation of laboratory tests can help determine whether nutritional deficiencies improve with dietary changes or supplementation. Based on the patient’s response and evolving needs, clinicians should be prepared to adjust the nutritional plan as necessary. Continuous encouragement and support will help patients maintain healthy eating habits over the long term, ultimately improving their fertility outcomes [56].

## Conclusions

In conclusion, the intricate relationship between nutritional deficiencies and subfertility underscores the importance of a holistic approach to reproductive health. Adequate intake of essential vitamins, minerals, and other nutrients is paramount for the proper functioning of the reproductive system in both men and women. Nutritional deficiencies can significantly disrupt hormonal balance, sperm and egg quality, and overall reproductive potential. Addressing these deficiencies through dietary interventions and supplementation can markedly improve fertility outcomes. The evidence presented in this review highlights the need for healthcare professionals to integrate nutritional assessment and guidance into fertility treatments. As research unravels the complex interactions between diet and fertility, personalized nutrition plans tailored to individual needs will become increasingly important. Ultimately, a comprehensive approach that includes nutritional optimization offers a promising avenue for enhancing reproductive health and aiding couples in achieving successful pregnancies.
